# Poly[[octa­aqua­tetra­kis­(μ_3_-pyridine-2,5-dicarboxyl­ato)copper(II)diytterbium(III)] monohydrate]

**DOI:** 10.1107/S1600536811014048

**Published:** 2011-04-22

**Authors:** Shie Fu Lush, Fwu Ming Shen

**Affiliations:** a Department of General Education Center, Yuanpei University, HsinChu 30015, Taiwan; b Department of Biotechnology, Yuanpei University, No. 306 Yuanpei St., HsinChu 30015, Taiwan

## Abstract

The asymmetric unit of the title heterometallic polymeric coordination compound, {[CuYb_2_(C_7_H_3_NO_4_)_4_(H_2_O)_8_]·H_2_O}_*n*_, contains one Cu^II^ cation located on an inversion center, a Yb^III^ cation, two pyridine-2,5-dicarboxyl­ate (pda) anions, four coordination water mol­ecules a disordered lattice water molecule, which is half-occupied and is located close to an inversion center. The Cu^II^ cation is *N*,*O*-chelated by two pda anions in the coordination basal plane and further coordinated by two carboxyl O atoms at the apical positions, with an elongated octa­hedral geometry. The Yb^III^ atom is eight-coordinated in a distorted square-anti­prismatic geometry formed by two carboxyl­ate O atoms from two pda anions, and is *N*,*O*-chelated by one pda anion and four coordinated water mol­ecules. The pda anions bridge adjacent Yb and Cu cations, forming a three-dimensional polymeric structure. The crystal structure features extensive O—H⋯O hydrogen bonds. π–π stacking is observed between parallel pyridine rings, the centroid–centroid distance being 3.843 (4) Å.

## Related literature

For related structures, see: Bai *et al.* (2008 ▶); Chi *et al.* (2009 ▶); Wang *et al.* (2009 ▶); Yue *et al.* (2007 ▶); Zhang *et al.* (2006 ▶). For structures in which the Cu atom displays an elongated octa­hedral geometry with a longer Cu—O bond, see: Chuang *et al.* (2008 ▶); Ghosh *et al.* (2004 ▶).
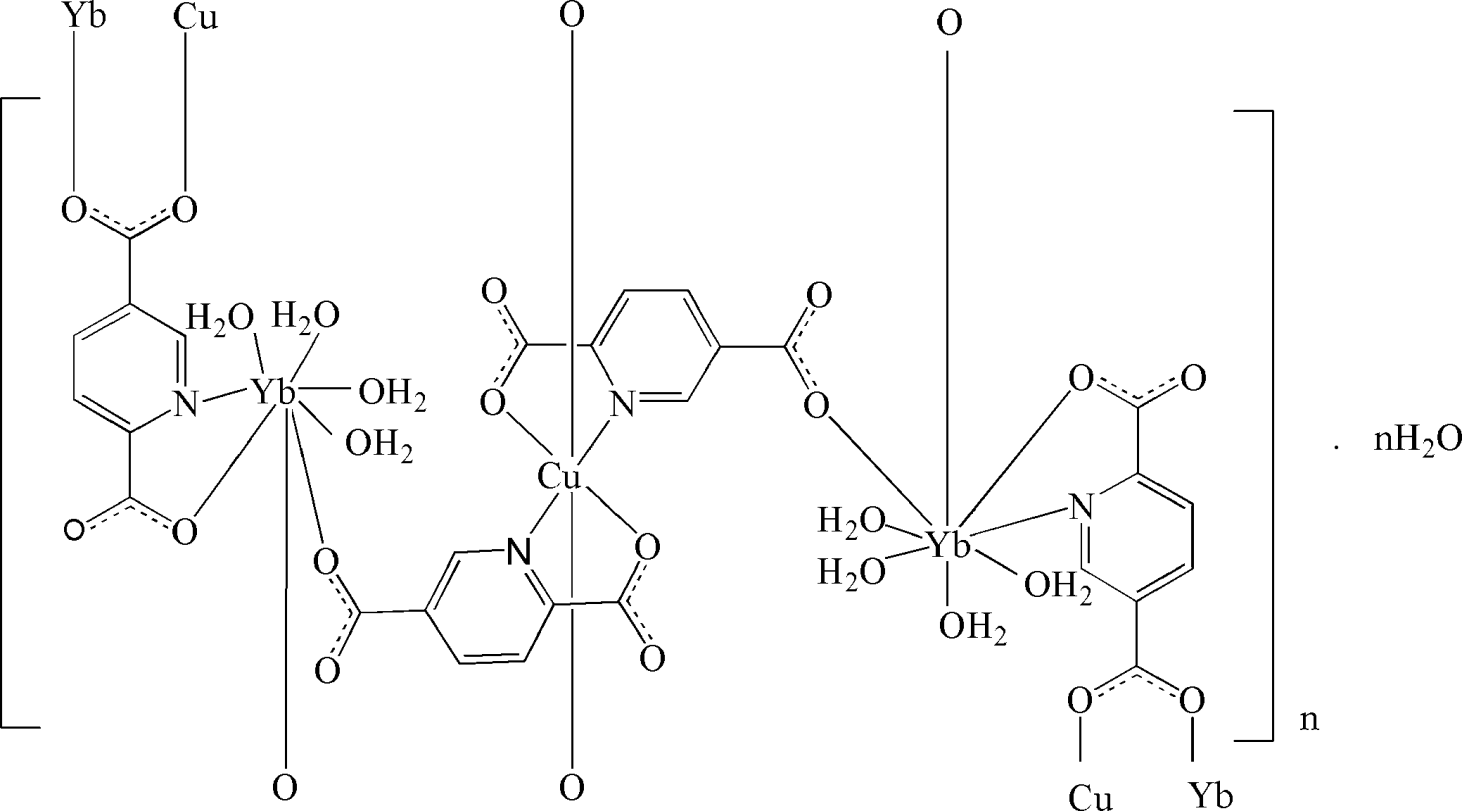


## Experimental

### 

#### Crystal data

[CuYb_2_(C_7_H_3_NO_4_)_4_(H_2_O)_8_]·H_2_O
*M* *_r_* = 1232.19
Triclinic, 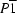

*a* = 7.7120 (5) Å
*b* = 9.2713 (6) Å
*c* = 13.2452 (9) Å
α = 75.529 (1)°
β = 76.216 (1)°
γ = 78.117 (1)°
*V* = 879.73 (10) Å^3^
*Z* = 1
Mo *K*α radiation
μ = 5.98 mm^−1^
*T* = 294 K
0.15 × 0.15 × 0.03 mm

#### Data collection

Bruker SMART CCD area-detector diffractometer
Absorption correction: multi-scan (*SADABS*; Bruker, 2001 ▶) *T*_min_ = 0.646, *T*_max_ = 0.984
7620 measured reflections
3150 independent reflections
3010 reflections with *I* > 2σ(*I*)
*R*_int_ = 0.036

#### Refinement

*R*[*F* ^2^ > 2σ(*F* ^2^)] = 0.044
*wR*(*F* ^2^) = 0.095
*S* = 1.18
3150 reflections
271 parameters
H-atom parameters constrained
Δρ_max_ = 2.78 e Å^−3^
Δρ_min_ = −2.60 e Å^−3^

### 

Data collection: *SMART* (Bruker, 2007 ▶); cell refinement: *SAINT* (Bruker, 2007 ▶); data reduction: *SAINT*; program(s) used to solve structure: *SHELXS97* (Sheldrick, 2008 ▶); program(s) used to refine structure: *SHELXL97* (Sheldrick, 2008 ▶); molecular graphics: *PLATON* (Spek, 2009 ▶); software used to prepare material for publication: *PLATON*.

## Supplementary Material

Crystal structure: contains datablocks global, I. DOI: 10.1107/S1600536811014048/xu5183sup1.cif

Structure factors: contains datablocks I. DOI: 10.1107/S1600536811014048/xu5183Isup2.hkl

Additional supplementary materials:  crystallographic information; 3D view; checkCIF report

## Figures and Tables

**Table 1:** Selected bond lengths (Å)

Yb1—N1	2.495 (7)
Yb1—O1	2.365 (6)
Yb1—O2	2.254 (7)
Yb1—O3	2.330 (8)
Yb1—O4	2.346 (7)
Yb1—O5	2.297 (5)
Yb1—O8^i^	2.297 (6)
Yb1—O9	2.334 (8)
Cu1—N2	1.985 (7)
Cu1—O7^ii^	2.641 (6)
Cu1—O11	1.944 (6)

Symmetry codes: (i) 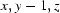; (ii) 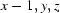.

**Table 2:** Hydrogen-bond geometry (Å, °)

*D*—H⋯*A*	*D*—H	H⋯*A*	*D*⋯*A*	*D*—H⋯*A*
O1—H1*A*⋯O12^iii^	0.81	1.96	2.757 (9)	165
O1—H1*B*⋯O11^iv^	0.82	2.02	2.782 (9)	153
O2—H2*A*⋯O10	0.88	1.88	2.670 (11)	149
O2—H2*B*⋯O5^v^	0.82	1.87	2.667 (9)	163
O3—H3*A*⋯O7^i^	0.84	1.82	2.607 (9)	155
O3—H3*B*⋯O13	0.82	1.95	2.73 (2)	159
O4—H4*A*⋯O10^vi^	0.82	1.95	2.760 (10)	167
O4—H4*B*⋯O6^vii^	0.82	1.98	2.802 (9)	179
O13—H13*A*⋯O10	0.87	2.20	3.03 (2)	161
O13—H13*B*⋯O12^i^	0.85	1.96	2.81 (2)	177

Symmetry codes: (i) 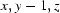; (iii) 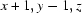; (iv) 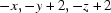; (v) 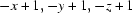; (vi) 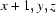; (vii) 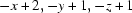.

**Fig. 1.**
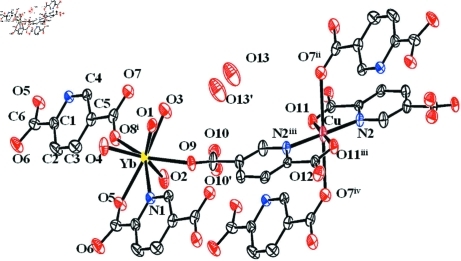
View of the title compound with the atom numbering scheme. Displacement ellipsoids for non-H atoms are drawn at the 50% probability level.H atoms have been omitted for clarity.[symmetry code:(i)x, y - 1, z; (ii) x - 1, y, z; (iii) -x - 1, -y + 2, -z + 2; (iv) -x, -y + 2, -z + 2].

**Fig. 2.**
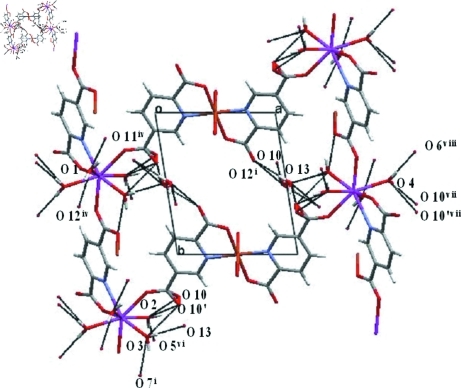
The molecular packing for the title compound, viewed along the c axis. Hydrogen bonds are shown as dashed lines.

## supplementary crystallographic information

### Comment

In recent year, many studies select pyridine-2,5-dicarboxylic acid as a bridging ligand, because it offers both N– and O-donors. Thus, the carboxylate group can bond to the lanthanide, while the nitrogen atom can bond to transition metal ions, allowing the possibility of 3 d-4f heterometallic coordination polymers (Zhang *et al.*, 2006; Yue *et al.*, 2007; Bai *et al.*, 2008; Chi *et al.*, 2009; Wang *et al.*, 2009).

Herein, we successfully prepared a heterometallic coordination polymer, [CuYb_2_(C_7_H_3_NO_4_)_4_(H_2_O)_8_.H_2_O]_n_, from a hydrothermal reaction. Fig. 1 shows the structure unit of the title complex, which contains one Cu^II^ and two Yb^III^ atoms, four pda ligands, eight coordinating and one non-coordinating water molecules. The Yb^III^ center is eight-coordinated [YbNO_3_(H_2_O)_4_] in a slightly distorted square-antiprismatic geometry formed by two carboxylate O atoms from two pda anions, N,*O*-chelated by one pda anion and four coordinated water molecules. One Cu^II^ atom is N,*O*-chelated by two pda anions in the coordination basal plane and coordinated by two carboxyl O atoms at the apical position with an elongated octahedral geometry (selected bond lengths are given in Table 1) (Ghosh *et al.*,2004; Chuang *et al.*, 2008). The molecular structure contains both Cu and Yb atoms, with pda ligands bridging the six coordinate Cu^II^ centers and eight coordinate Yb^III^ centers to form a three-dimensional net structure.

The crystal structure contains the extensive O—H···O (shown as Fig. 2 and Table 2). π···π stackings are present in the crystal structure, the shortest centroid distance between parallel pyridine rings *Cg*5^iv^···*Cg*5((N2/C8—C12) is 3.843 (4) Å, respectively [symmetry code:(iv)=-*X*, 2-*Y*,2-*Z*].

### Experimental

A solution of Cu(OAc)_2_.H_2_O (0.0205 g, 0.10 mmol), Yb_2_O_3_ (0.0199 g, 0.050 mmol) and 2,5-pyridinedicarboxylic acid (0.0343 g, 0.20 mmol) were mixed in 10 ml deionized water. After stirring half an hour, the mixture was placed in 23 ml Teflon-lined reactor. After heating for four days at 418 K, the mixture was cooling to room-temperature. Green block-like crystals were isolated in 42% yield (based on Yb).

### Refinement

Water H atoms were fixed in chemical sensible positions, their thermal parameters were fixed as 0.08 Å^2^. Other H atoms were positioned geometrically with C—H = 0.93 Å and refined using a riding model, *U*_iso_(H) = 1.2*U*_eq_(C).

### Crystal data

**Table:**

[CuYb_2_(C_7_H_3_NO_4_)_4_(H_2_O)_8_]·H_2_O	*Z* = 1
*M**_r_* = 1232.19	*F*(000) = 595
Triclinic, *P*1	*D*_x_ = 2.326 Mg m^−^^3^
Hall symbol: -P 1	Mo *K*α radiation, λ = 0.71073 Å
*a* = 7.7120 (5) Å	Cell parameters from 5946 reflections
*b* = 9.2713 (6) Å	θ = 2.5–25.0°
*c* = 13.2452 (9) Å	µ = 5.98 mm^−^^1^
α = 75.529 (1)°	*T* = 294 K
β = 76.216 (1)°	Tabular, green
γ = 78.117 (1)°	0.15 × 0.15 × 0.03 mm
*V* = 879.73 (10) Å^3^	

### Data collection

**Table:**

Bruker SMART CCD area-detector diffractometer	3150 independent reflections
Radiation source: fine-focus sealed tube	3010 reflections with *I* > 2σ(*I*)
graphite	*R*_int_ = 0.036
Detector resolution: 9 pixels mm^-1^	θ_max_ = 25.2°, θ_min_ = 1.6°
φ and ω scans	*h* = −9→9
Absorption correction: multi-scan (*SADABS*; Bruker, 2001)	*k* = −11→10
*T*_min_ = 0.646, *T*_max_ = 0.984	*l* = −15→15
7620 measured reflections	

### Refinement

**Table:**

Refinement on *F*^2^	Primary atom site location: structure-invariant direct methods
Least-squares matrix: full	Secondary atom site location: difference Fourier map
*R*[*F*^2^ > 2σ(*F*^2^)] = 0.044	Hydrogen site location: inferred from neighbouring sites
*wR*(*F*^2^) = 0.095	H-atom parameters constrained
*S* = 1.18	*w* = 1/[σ^2^(*F*_o_^2^) + (0.0263*P*)^2^ + 10.305*P*] where *P* = (*F*_o_^2^ + 2*F*_c_^2^)/3
3150 reflections	(Δ/σ)_max_ = 0.005
271 parameters	Δρ_max_ = 2.78 e Å^−^^3^
0 restraints	Δρ_min_ = −2.60 e Å^−^^3^

### Special details

**Table:**

Geometry. Bond distances, angles *etc*. have been calculated using the rounded fractional coordinates. All su's are estimated from the variances of the (full) variance-covariance matrix. The cell e.s.d.'s are taken into account in the estimation of distances, angles and torsion angles
Refinement. Refinement on *F*^2^ for ALL reflections except those flagged by the user for potential systematic errors. Weighted *R*-factors *wR* and all goodnesses of fit *S* are based on *F*^2^, conventional *R*-factors *R* are based on *F*, with *F* set to zero for negative *F*^2^. The observed criterion of *F*^2^ > σ(*F*^2^) is used only for calculating -*R*-factor-obs *etc*. and is not relevant to the choice of reflections for refinement. *R*-factors based on *F*^2^ are statistically about twice as large as those based on *F*, and *R*-factors based on ALL data will be even larger.

### Fractional atomic coordinates and isotropic or equivalent isotropic displacement parameters (Å^2^)

**Table:**

	*x*	*y*	*z*	*U*_iso_*/*U*_eq_	Occ. (<1)
Yb1	0.54822 (5)	0.55527 (4)	0.70031 (3)	0.0202 (1)	
Cu1	−0.50000	1.00000	1.00000	0.0294 (5)	
O1	0.6110 (8)	0.6005 (7)	0.8550 (5)	0.0303 (17)	
O2	0.3316 (9)	0.5549 (8)	0.6123 (6)	0.040 (2)	
O3	0.3630 (10)	0.4196 (7)	0.8421 (6)	0.045 (2)	
O4	0.8540 (8)	0.4781 (7)	0.7109 (5)	0.036 (2)	
O5	0.6911 (8)	0.6043 (6)	0.5249 (4)	0.0292 (19)	
O6	0.8886 (10)	0.7235 (7)	0.3948 (5)	0.044 (3)	
O7	0.4698 (8)	1.1486 (6)	0.8054 (5)	0.0300 (19)	
O8	0.6197 (8)	1.3112 (6)	0.6768 (5)	0.033 (2)	
O9	0.3050 (9)	0.7337 (9)	0.7547 (7)	0.0534 (19)	
O10	0.0479 (9)	0.6787 (9)	0.7398 (7)	0.0534 (19)	
O11	−0.4478 (8)	1.1832 (6)	1.0248 (5)	0.0300 (17)	
O12	−0.2411 (9)	1.3353 (7)	0.9741 (5)	0.034 (2)	
N1	0.6426 (9)	0.8097 (7)	0.6374 (5)	0.0213 (19)	
N2	−0.2416 (9)	0.9775 (8)	0.9285 (5)	0.024 (2)	
C1	0.7354 (11)	0.8421 (9)	0.5374 (7)	0.026 (3)	
C2	0.7878 (14)	0.9813 (10)	0.4904 (7)	0.038 (3)	
C3	0.7363 (14)	1.0958 (10)	0.5471 (8)	0.037 (3)	
C4	0.6381 (11)	1.0647 (9)	0.6510 (7)	0.023 (2)	
C5	0.5969 (11)	0.9204 (8)	0.6927 (6)	0.021 (2)	
C6	0.7773 (12)	0.7156 (10)	0.4794 (7)	0.029 (3)	
C7	0.5715 (11)	1.1839 (9)	0.7168 (7)	0.025 (3)	
C8	−0.1440 (12)	0.8674 (10)	0.8796 (7)	0.030 (3)	
C9	0.0322 (11)	0.8752 (9)	0.8247 (7)	0.026 (3)	
C10	0.1051 (11)	1.0033 (9)	0.8160 (7)	0.026 (2)	
C11	0.0035 (11)	1.1185 (9)	0.8637 (7)	0.027 (3)	
C12	−0.1703 (11)	1.1007 (9)	0.9198 (6)	0.023 (2)	
C13	0.1376 (12)	0.7481 (11)	0.7720 (8)	0.035 (3)	
C14	−0.2915 (11)	1.2177 (9)	0.9759 (6)	0.022 (2)	
O13	0.035 (3)	0.497 (2)	0.9652 (15)	0.073 (8)	0.500
H1A	0.66390	0.53210	0.89360	0.0800*	
H1B	0.53470	0.64950	0.89350	0.0800*	
H2A	0.21830	0.57560	0.64460	0.0800*	
H2B	0.33500	0.49070	0.57830	0.0800*	
H2C	0.85660	0.99810	0.42180	0.0450*	
H3A	0.36350	0.33120	0.83550	0.0800*	
H3B	0.28010	0.43640	0.89150	0.0800*	
H3C	0.76680	1.19130	0.51630	0.0450*	
H4A	0.91280	0.52870	0.72840	0.0800*	
H4B	0.92920	0.41980	0.67930	0.0800*	
H5A	0.53430	0.89870	0.76270	0.0250*	
H8A	−0.19600	0.78400	0.88270	0.0350*	
H10A	0.22200	1.01180	0.77820	0.0300*	
H11A	0.05020	1.20550	0.85840	0.0320*	
H13A	0.01660	0.56250	0.90790	0.0800*	0.500
H13B	−0.04850	0.44950	0.96530	0.0800*	0.500

### Atomic displacement parameters (Å^2^)

**Table:**

	*U*^11^	*U*^22^	*U*^33^	*U*^12^	*U*^13^	*U*^23^
Yb1	0.0224 (2)	0.0149 (2)	0.0232 (2)	−0.0037 (1)	0.0008 (1)	−0.0086 (1)
Cu1	0.0187 (7)	0.0260 (8)	0.0449 (9)	−0.0005 (6)	0.0028 (6)	−0.0211 (7)
O1	0.035 (3)	0.026 (3)	0.029 (3)	0.007 (3)	−0.006 (3)	−0.014 (3)
O2	0.029 (3)	0.049 (4)	0.052 (4)	−0.003 (3)	−0.004 (3)	−0.036 (3)
O3	0.056 (5)	0.021 (3)	0.045 (4)	−0.006 (3)	0.016 (3)	−0.009 (3)
O4	0.025 (3)	0.039 (4)	0.050 (4)	0.000 (3)	−0.003 (3)	−0.028 (3)
O5	0.043 (4)	0.024 (3)	0.024 (3)	−0.013 (3)	0.004 (3)	−0.015 (2)
O6	0.058 (5)	0.030 (4)	0.037 (4)	−0.016 (3)	0.022 (3)	−0.016 (3)
O7	0.041 (4)	0.019 (3)	0.030 (3)	−0.007 (3)	−0.001 (3)	−0.009 (2)
O8	0.037 (4)	0.017 (3)	0.046 (4)	−0.011 (3)	0.005 (3)	−0.014 (3)
O9	0.026 (3)	0.061 (3)	0.088 (4)	−0.008 (2)	0.006 (3)	−0.058 (3)
O10	0.026 (3)	0.061 (3)	0.088 (4)	−0.008 (2)	0.006 (3)	−0.058 (3)
O11	0.030 (3)	0.025 (3)	0.035 (3)	0.000 (3)	0.000 (3)	−0.016 (3)
O12	0.036 (4)	0.020 (3)	0.048 (4)	−0.002 (3)	−0.008 (3)	−0.013 (3)
N1	0.024 (4)	0.017 (3)	0.023 (3)	−0.004 (3)	0.001 (3)	−0.009 (3)
N2	0.021 (4)	0.023 (4)	0.030 (4)	0.000 (3)	−0.004 (3)	−0.014 (3)
C1	0.021 (4)	0.024 (4)	0.030 (5)	0.000 (3)	−0.002 (3)	−0.005 (4)
C2	0.050 (6)	0.031 (5)	0.030 (5)	−0.014 (4)	0.010 (4)	−0.012 (4)
C3	0.052 (6)	0.020 (5)	0.037 (5)	−0.011 (4)	0.003 (4)	−0.007 (4)
C4	0.023 (4)	0.018 (4)	0.031 (4)	−0.005 (3)	−0.005 (3)	−0.011 (3)
C5	0.023 (4)	0.016 (4)	0.026 (4)	−0.003 (3)	−0.004 (3)	−0.008 (3)
C6	0.031 (5)	0.027 (5)	0.027 (5)	−0.011 (4)	−0.006 (4)	0.001 (4)
C7	0.024 (4)	0.019 (4)	0.036 (5)	0.000 (3)	−0.009 (4)	−0.012 (4)
C8	0.025 (4)	0.028 (5)	0.040 (5)	−0.005 (4)	−0.002 (4)	−0.019 (4)
C9	0.026 (5)	0.024 (4)	0.029 (4)	−0.001 (4)	−0.004 (4)	−0.012 (4)
C10	0.021 (4)	0.025 (4)	0.029 (4)	−0.001 (3)	−0.001 (3)	−0.008 (4)
C11	0.028 (5)	0.019 (4)	0.032 (5)	−0.001 (3)	−0.006 (4)	−0.004 (3)
C12	0.026 (4)	0.018 (4)	0.021 (4)	0.001 (3)	−0.004 (3)	−0.004 (3)
C13	0.021 (5)	0.032 (5)	0.058 (6)	0.001 (4)	−0.005 (4)	−0.028 (5)
C14	0.027 (4)	0.020 (4)	0.019 (4)	0.002 (3)	−0.004 (3)	−0.007 (3)
O13	0.061 (12)	0.087 (13)	0.076 (14)	−0.033 (10)	0.026 (9)	−0.047 (12)

### Geometric parameters (Å, °)

**Table:**

Yb1—N1	2.495 (7)	O3—H3A	0.8400
Yb1—O1	2.365 (6)	O4—H4A	0.8200
Yb1—O2	2.254 (7)	O4—H4B	0.8200
Yb1—O3	2.330 (8)	O13—H13B	0.8500
Yb1—O4	2.346 (7)	O13—H13A	0.8700
Yb1—O5	2.297 (5)	N1—C1	1.340 (11)
Yb1—O8^i^	2.297 (6)	N1—C5	1.348 (10)
Yb1—O9	2.334 (8)	N2—C12	1.335 (11)
Cu1—N2	1.985 (7)	N2—C8	1.345 (12)
Cu1—N2^ii^	1.985 (7)	C1—C6	1.499 (12)
Cu1—O7^iii^	2.641 (6)	C1—C2	1.382 (13)
Cu1—O7^iv^	2.641 (6)	C2—C3	1.390 (13)
Cu1—O11	1.944 (6)	C3—C4	1.395 (13)
Cu1—O11^ii^	1.944 (6)	C4—C7	1.510 (12)
O5—C6	1.285 (11)	C4—C5	1.385 (11)
O6—C6	1.235 (11)	C8—C9	1.387 (13)
O7—C7	1.255 (11)	C9—C10	1.384 (12)
O8—C7	1.259 (10)	C9—C13	1.511 (13)
O9—C13	1.241 (12)	C10—C11	1.382 (12)
O10—C13	1.239 (13)	C11—C12	1.390 (12)
O11—C14	1.288 (11)	C12—C14	1.502 (12)
O12—C14	1.224 (11)	C2—H2C	0.9300
O1—H1A	0.8100	C3—H3C	0.9300
O1—H1B	0.8200	C5—H5A	0.9300
O2—H2A	0.8800	C8—H8A	0.9300
O2—H2B	0.8200	C10—H10A	0.9300
O3—H3B	0.8200	C11—H11A	0.9300
			
O1—Yb1—O2	145.9 (2)	H3A—O3—H3B	107.00
O1—Yb1—O3	74.7 (2)	Yb1—O3—H3B	138.00
O1—Yb1—O4	68.0 (2)	H4A—O4—H4B	105.00
O1—Yb1—O5	132.0 (2)	Yb1—O4—H4B	128.00
O1—Yb1—O9	75.8 (3)	Yb1—O4—H4A	123.00
O1—Yb1—N1	77.2 (2)	H13A—O13—H13B	93.00
O1—Yb1—O8^i^	116.9 (2)	Yb1—N1—C5	125.5 (5)
O2—Yb1—O3	82.9 (3)	C1—N1—C5	117.5 (7)
O2—Yb1—O4	145.5 (2)	Yb1—N1—C1	116.9 (5)
O2—Yb1—O5	76.6 (2)	Cu1—N2—C12	111.4 (6)
O2—Yb1—O9	73.8 (3)	C8—N2—C12	119.2 (7)
O2—Yb1—N1	106.8 (2)	Cu1—N2—C8	128.8 (6)
O2—Yb1—O8^i^	80.5 (2)	C2—C1—C6	122.2 (8)
O3—Yb1—O4	111.2 (2)	N1—C1—C6	114.8 (7)
O3—Yb1—O5	151.4 (2)	N1—C1—C2	123.0 (8)
O3—Yb1—O9	74.9 (3)	C1—C2—C3	119.1 (9)
O3—Yb1—N1	140.3 (2)	C2—C3—C4	118.7 (9)
O3—Yb1—O8^i^	75.0 (2)	C5—C4—C7	119.7 (8)
O4—Yb1—O5	77.5 (2)	C3—C4—C7	122.1 (8)
O4—Yb1—O9	139.4 (3)	C3—C4—C5	118.1 (8)
O4—Yb1—N1	82.8 (2)	N1—C5—C4	123.5 (7)
O4—Yb1—O8^i^	73.7 (2)	O6—C6—C1	119.9 (8)
O5—Yb1—O9	117.1 (3)	O5—C6—C1	115.0 (8)
O5—Yb1—N1	66.0 (2)	O5—C6—O6	125.2 (8)
O5—Yb1—O8^i^	82.0 (2)	O7—C7—C4	117.2 (7)
O9—Yb1—N1	71.5 (3)	O8—C7—C4	117.0 (8)
O8^i^—Yb1—O9	142.3 (3)	O7—C7—O8	125.7 (8)
O8^i^—Yb1—N1	143.8 (2)	N2—C8—C9	121.7 (8)
O11—Cu1—N2	83.1 (3)	C8—C9—C13	119.9 (8)
O7^iv^—Cu1—O11	87.9 (2)	C10—C9—C13	121.4 (8)
O11—Cu1—O11^ii^	180.00	C8—C9—C10	118.7 (8)
O11—Cu1—N2^ii^	96.9 (3)	C9—C10—C11	119.8 (8)
O7^iii^—Cu1—O11	92.1 (2)	C10—C11—C12	118.2 (8)
O7^iv^—Cu1—N2	80.1 (2)	N2—C12—C14	115.0 (7)
O11^ii^—Cu1—N2	96.9 (3)	N2—C12—C11	122.4 (8)
N2—Cu1—N2^ii^	180.00	C11—C12—C14	122.7 (8)
O7^iii^—Cu1—N2	99.9 (2)	O10—C13—C9	116.1 (9)
O7^iv^—Cu1—O11^ii^	92.1 (2)	O9—C13—O10	126.0 (10)
O7^iv^—Cu1—N2^ii^	99.9 (2)	O9—C13—C9	117.4 (9)
O7^iv^—Cu1—O7^iii^	180.00	O12—C14—C12	121.1 (8)
O11^ii^—Cu1—N2^ii^	83.1 (3)	O11—C14—C12	114.8 (7)
O7^iii^—Cu1—O11^ii^	87.9 (2)	O11—C14—O12	124.1 (8)
O7^iii^—Cu1—N2^ii^	80.1 (2)	C1—C2—H2C	120.00
Yb1—O5—C6	125.3 (5)	C3—C2—H2C	120.00
Cu1^v^—O7—C7	138.2 (6)	C4—C3—H3C	121.00
Yb1^vi^—O8—C7	140.4 (6)	C2—C3—H3C	121.00
Yb1—O9—C13	136.8 (7)	N1—C5—H5A	118.00
Cu1—O11—C14	114.7 (5)	C4—C5—H5A	118.00
H1A—O1—H1B	107.00	C9—C8—H8A	119.00
Yb1—O1—H1A	119.00	N2—C8—H8A	119.00
Yb1—O1—H1B	121.00	C9—C10—H10A	120.00
Yb1—O2—H2A	117.00	C11—C10—H10A	120.00
Yb1—O2—H2B	124.00	C10—C11—H11A	121.00
H2A—O2—H2B	107.00	C12—C11—H11A	121.00
Yb1—O3—H3A	113.00		
			
O1—Yb1—O5—C6	−31.3 (8)	Cu1^v^—O7—C7—C4	80.0 (10)
O2—Yb1—O5—C6	127.4 (7)	Yb1^vi^—O8—C7—O7	−13.3 (16)
O3—Yb1—O5—C6	172.9 (7)	Yb1^vi^—O8—C7—C4	165.0 (6)
O4—Yb1—O5—C6	−75.6 (7)	Yb1—O9—C13—O10	−18.5 (18)
O9—Yb1—O5—C6	63.8 (7)	Yb1—O9—C13—C9	170.0 (7)
N1—Yb1—O5—C6	12.0 (7)	Cu1—O11—C14—O12	173.2 (7)
O8^i^—Yb1—O5—C6	−150.5 (7)	Cu1—O11—C14—C12	−7.1 (9)
O1—Yb1—O9—C13	−129.3 (11)	Yb1—N1—C1—C2	176.5 (7)
O2—Yb1—O9—C13	35.3 (10)	Yb1—N1—C1—C6	−3.0 (10)
O3—Yb1—O9—C13	−51.6 (11)	C5—N1—C1—C2	1.2 (13)
O4—Yb1—O9—C13	−156.7 (9)	C5—N1—C1—C6	−178.3 (8)
O5—Yb1—O9—C13	100.5 (11)	Yb1—N1—C5—C4	−173.7 (6)
N1—Yb1—O9—C13	149.8 (11)	C1—N1—C5—C4	1.1 (13)
O8^i^—Yb1—O9—C13	−13.5 (13)	Cu1—N2—C8—C9	174.0 (6)
O1—Yb1—N1—C1	145.0 (6)	C12—N2—C8—C9	3.2 (13)
O1—Yb1—N1—C5	−40.1 (7)	Cu1—N2—C12—C11	−173.7 (7)
O2—Yb1—N1—C1	−70.1 (6)	Cu1—N2—C12—C14	6.8 (8)
O2—Yb1—N1—C5	104.8 (7)	C8—N2—C12—C11	−1.4 (12)
O3—Yb1—N1—C1	−169.3 (6)	C8—N2—C12—C14	179.1 (7)
O3—Yb1—N1—C5	5.6 (9)	N1—C1—C2—C3	−2.8 (15)
O4—Yb1—N1—C1	76.0 (6)	C6—C1—C2—C3	176.7 (9)
O4—Yb1—N1—C5	−109.2 (7)	N1—C1—C6—O5	12.5 (12)
O5—Yb1—N1—C1	−3.5 (6)	N1—C1—C6—O6	−167.0 (8)
O5—Yb1—N1—C5	171.4 (7)	C2—C1—C6—O5	−167.0 (9)
O9—Yb1—N1—C1	−135.9 (7)	C2—C1—C6—O6	13.5 (14)
O9—Yb1—N1—C5	39.0 (7)	C1—C2—C3—C4	2.0 (15)
O8^i^—Yb1—N1—C1	26.7 (8)	C2—C3—C4—C5	0.1 (14)
O8^i^—Yb1—N1—C5	−158.4 (6)	C2—C3—C4—C7	−177.9 (9)
O1—Yb1—O8^i^—C7^i^	68.2 (9)	C3—C4—C5—N1	−1.8 (14)
O2—Yb1—O8^i^—C7^i^	−80.8 (9)	C7—C4—C5—N1	176.3 (8)
O3—Yb1—O8^i^—C7^i^	4.4 (9)	C3—C4—C7—O7	173.3 (9)
O4—Yb1—O8^i^—C7^i^	122.3 (9)	C3—C4—C7—O8	−5.1 (13)
O5—Yb1—O8^i^—C7^i^	−158.4 (9)	C5—C4—C7—O7	−4.7 (13)
O9—Yb1—O8^i^—C7^i^	−33.7 (11)	C5—C4—C7—O8	176.9 (8)
N1—Yb1—O8^i^—C7^i^	173.9 (8)	N2—C8—C9—C10	−3.3 (13)
N2—Cu1—O11—C14	8.6 (6)	N2—C8—C9—C13	179.3 (8)
O7^iv^—Cu1—O11—C14	−71.7 (6)	C8—C9—C10—C11	1.4 (13)
N2^ii^—Cu1—O11—C14	−171.4 (6)	C13—C9—C10—C11	178.8 (8)
O7^iii^—Cu1—O11—C14	108.3 (6)	C8—C9—C13—O9	−157.1 (9)
O11—Cu1—N2—C8	−179.6 (8)	C8—C9—C13—O10	30.5 (13)
O11—Cu1—N2—C12	−8.3 (5)	C10—C9—C13—O9	25.5 (14)
O7^iv^—Cu1—N2—C8	−90.5 (7)	C10—C9—C13—O10	−146.9 (10)
O7^iv^—Cu1—N2—C12	80.8 (5)	C9—C10—C11—C12	0.3 (13)
O11^ii^—Cu1—N2—C8	0.4 (8)	C10—C11—C12—N2	−0.3 (13)
O11^ii^—Cu1—N2—C12	171.7 (5)	C10—C11—C12—C14	179.2 (8)
O7^iii^—Cu1—N2—C8	89.5 (7)	N2—C12—C14—O11	0.0 (10)
O7^iii^—Cu1—N2—C12	−99.2 (5)	N2—C12—C14—O12	179.7 (8)
Yb1—O5—C6—O6	161.7 (7)	C11—C12—C14—O11	−179.5 (8)
Yb1—O5—C6—C1	−17.8 (11)	C11—C12—C14—O12	0.2 (12)
Cu1^v^—O7—C7—O8	−101.7 (10)		

Symmetry codes: (i) *x*, *y*−1, *z*; (ii) −*x*−1, −*y*+2, −*z*+2; (iii) −*x*, −*y*+2, −*z*+2; (iv) *x*−1, *y*, *z*; (v) *x*+1, *y*, *z*; (vi) *x*, *y*+1, *z*.

### Hydrogen-bond geometry (Å, °)

**Table:**

*D*—H···*A*	*D*—H	H···*A*	*D*···*A*	*D*—H···*A*
O1—H1A···O12^vii^	0.81	1.96	2.757 (9)	165
O1—H1B···O11^iii^	0.82	2.02	2.782 (9)	153
O2—H2A···O10	0.88	1.88	2.670 (11)	149
O2—H2B···O5^viii^	0.82	1.87	2.667 (9)	163
O3—H3A···O7^i^	0.84	1.82	2.607 (9)	155
O3—H3B···O13	0.82	1.95	2.73 (2)	159
O4—H4A···O10^v^	0.82	1.95	2.760 (10)	167
O4—H4B···O6^ix^	0.82	1.98	2.802 (9)	179
O13—H13A···O10	0.87	2.20	3.03 (2)	161
O13—H13B···O12^i^	0.85	1.96	2.81 (2)	177

Symmetry codes: (vii) *x*+1, *y*−1, *z*; (iii) −*x*, −*y*+2, −*z*+2; (viii) −*x*+1, −*y*+1, −*z*+1; (i) *x*, *y*−1, *z*; (v) *x*+1, *y*, *z*; (ix) −*x*+2, −*y*+1, −*z*+1.
